# Effect of high contents of dietary animal-derived protein or carbohydrates on canine faecal microbiota

**DOI:** 10.1186/1746-6148-8-90

**Published:** 2012-06-26

**Authors:** Ingrid Hang, Teemu Rinttila, Jürgen Zentek, Anu Kettunen, Susanna Alaja, Juha Apajalahti, Jaana Harmoinen, Willem M de Vos, Thomas Spillmann

**Affiliations:** 1 Department of Equine and Small Animal Medicine, University of Helsinki, 57, Finland; 2 Alimetrics Ltd, Espoo, Finland; 3 Institute of Animal Nutrition, Section of Veterinary Medicine, Free University Berlin, Germany; 4 Department of Basic Veterinary Sciences, University of Helsinki, Finland; and Laboratory of Microbiology, Wageningen University, The Netherlands

## Abstract

**Background:**

Considerable evidence suggests that food impacts both the gastro-intestinal (GI) function and the microbial ecology of the canine GI tract. The aim of this study was to evaluate the influence of high-carbohydrate (HC), high-protein (HP) and dry commercial (DC) diets on the canine colonic microbiota in Beagle dogs. Diets were allocated according to the Graeco-Latin square design. For this purpose, microbial DNA was isolated from faecal samples and separated by density gradient centrifugation, resulting in specific profiling based on the guanine-cytosine content (%G + C). In addition, 16 S rRNA gene amplicons were obtained from the most abundant %G + C peaks and analysed by sequence analysis, producing a total of 720 non-redundant sequences (240 sequences per diet).

**Results:**

The DC diet sample showed high abundance of representatives of the orders *Clostridiales, Lactobacillales, Coriobacteriales* and *Bacteroidales*. Sequence diversity was highest for DC diet samples and included representatives of the orders *Lactobacillales* and *Bacteroidales*, which were not detected in samples from the HP and HC diets. These latter two diets also had reduced levels of representatives of the family *Lachnospiraceae*, specifically Clostridial cluster XIVa. The HC diet favoured representatives of the order *Erysipelotrichales*, more specifically the Clostridial cluster XVIII, while the HP diet favoured representatives of the order *Fusobacteriales*.

**Conclusions:**

This study detected *Coriobacteriales* in dog faeces, possibly due to the non-selective nature of the %G + C profiling method used in combination with sequencing. Moreover, our work demonstrates that the effect of diet on faecal microbiota can be explained based on the metabolic properties of the detected microbial taxa.

## Background

The microbial ecology of the canine gastro-intestinal (GI) tract is a rapidly expanding research area in veterinary medicine. The intestinal tract harbours a large number of prokaryotes, mainly bacteria, which exceed the number of host cells. Complex interactions exist between the eukaryotic and prokaryotic components; the latter are important in maintaining the health of the former by playing a vital role in the normal nutritional, physiological, immunological and protective functions of the host [1]. The amount and form of food, feeding frequency and diet composition are known to have important effects on GI function. Both nutrients and non-nutritional dietary components influence gut health in terms of intestinal microbiota [2]. Alterations in the intestinal microbiota or aberrations in immune responses to its components are hypothesized to play a crucial role in the pathogenesis of enteropathies (e.g., inflammatory bowel disease, dietary intolerance, sensitivity and allergy) [1]. An important focus of canine research has been the effect of different diets on satiety, faecal consistency and quantity of *Clostridium perfringens* in faeces [3,4]. Recently, two studies have been published about the fluctuations in canine faecal bacterial populations caused by dietary changes [5,6]. Human studies using conventional culturing techniques have indicated that the protein and fat content of the diet as well as the nature of the carbohydrates (simple sugars vs. complex carbohydrates) does affect microbiota composition and activity [7]. Studies in chickens, rats and mice support the hypothesis that the intestinal microbiota can be modified by diet [8-10].

To date, seven bacterial groups (*Bacteroides, Clostridium, Lactobacillus, Bifidobacterium, Fusobacterium, Enterobacteriaceae* and *Coriobacterium)* in five predominant phyla (*Firmicutes, Fusobacteria, Bacteroidetes, Proteobacteria* and *Actinobacteria*) have been identified from different parts of the canine intestine using culture techniques and/or various molecular methods [6,11-13].

Previously, the main technique for studying the canine GI tract microbial community structure has been the cultivation of bacteria from intestinal contents [12,14]. However, the cultivable bacteria in the animal intestinal tract represent only a fraction of the microbes actually present in the gut, obviating the need for high-throughput molecular approaches, many of which rely on the sequence of the bacterial 16 S rRNA genes that serve as phylogenetic markers [15]. Although these molecular techniques have apparent benefits over the traditional bacterial culturing, they possess several potential pitfalls that must be taken into account. The main issues for quantification of nucleic acids in complex communities relate to the repeatability of DNA extraction from different bacteria or sample types with constant efficiency and sufficient purity [16]. The profiling of guanine-cytosine content (%G + C) is a technique for initial investigation of bacterial populations of previously unknown structure. The great benefit of this method over other DNA-based methods is its lack of dependence on any *a priori* information about the bacteria being analysed. Moreover, no PCR amplification is required, which is known to introduce artefacts with an increasing number of cycles [1,17]. This technique relies on the separation of chromosomal DNA of various bacterial species by density gradient centrifugation and yields a profile based on their characteristic guanine-cytosine content [18]. The individual G + C fractions from the pool of bacterial chromosomal DNA with any G-C content can be collected for subsequent detailed analyses, including cloning and sequencing of the 16 S rRNA genes [18,19]. This approach been successfully employed to study microbial community structures in a variety of environments, such as soils, or the GI tracts of humans or different animals [18-22].

As mentioned above, specific interactions between diets differing in macronutrient content and microbiota composition have rarely been investigated with 16 S rDNA-based molecular tools in dogs. Therefore, the aim of the current study was to investigate the alterations in canine intestinal microbiota due to dietary changes by applying %G + C profiling for total community analysis, followed by sequencing of relevant fractions of nearly complete 16 S rRNA gene fragments.

## Results

Graeco-Latin square design was used to evaluate the influence of high-carbohydrate (HC), high-protein (HP) and dry commercial (DC) diets on the colonic microbiota of five Beagle dogs. Isolated bacterial DNA from canine faecal samples obtained during the feeding of one of the three specialized diets was used for %G + C profiling and sequencing of valid fractions (referred to herein as fractions 5, 10 and 14) from the %G + C profile. Fractions 5, 10 and 14 corresponded to %G + C ranges of 27–32, 46.5-51.5 and 62–67, respectively.

**Figure 1:**
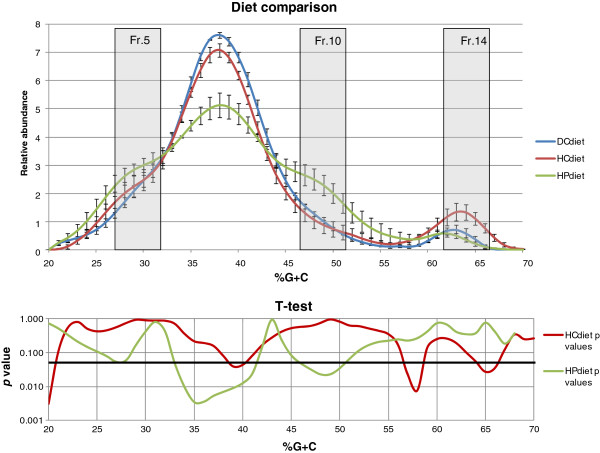
%G + C profiles and fractions 5, 10 and 14 of DC, HP and HC diets.

**%G + C profiling of DNA samples** - The DC diet faecal samples displayed a significantly higher abundance of microbes with %G + C between 33 and 41 than the HP diet samples (p = 0.03) (Figure 1). Moreover, samples from the HP diet contained a peak at %G + C between 46 and 50, which was completely lacking from the DC diet samples (p = 0.02, Figure 1). A low %G + C peak was present in the HP diet and lacking in DC diet samples in the %G + C range of 25–29 (p = 0.05, Figure 1).

No major differences between the DC and HC diet samples were observed. However, the profiles were significantly different at %G + C 39–40, 57–58 and 65–66 (p < 0.05, Figure 1).

To illustrate the difference between the HP and HC diet, we also carried out a direct comparison between these two diets. As expected, considerable differences were observed in the average %G + C profiles (Figure 1). The HC diet resulted in a significant higher abundance of microbes with %G + C between 33 and 40 than the HP diet (p < 0.01, Figure 1). The peak present in the HP diet samples at %G + C between 46 and 50 was completely absent in the HC diet samples (p = 0.02, Figure 1). The HC diet samples favoured bacteria with %G + C higher than 60 (p < 0.05, Figure 1).

### **Phylogenetic analysis of fraction 5 sequences**

**Figure 2:**
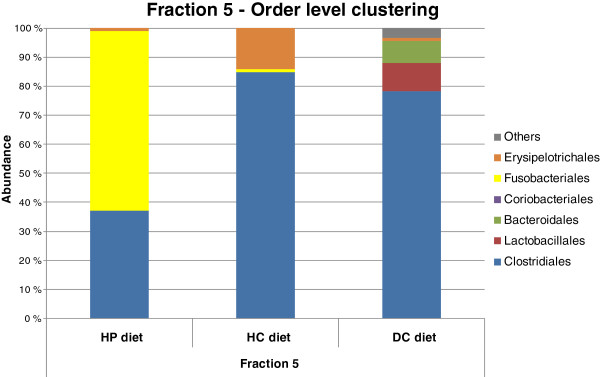
Phylogenetic analysis of fraction 5 sequences with DC, HP and HC diets.

Ninety-six sequences per sample were obtained from %G + C fraction 5. *Clostridiales* was the most representative bacterial order in DNA obtained from faecal samples of dogs fed DC and HC diets (78 % and 85 % of clones, respectively). Overall, the proportion of *Clostridiales* sequences in the HP diet sample was much lower (37 %, Figure 2). This difference was statistically significant with order-level library comparison analysis between the HP and DC diet samples as well as between the HC and DC diet samples (p < 0.01).

At family-level classification, the *Clostridiales* clones from DC diet samples were distributed into two main bacterial families, namely *Lachnospiraceae* (72 % of *Clostridiales* clones) and *Peptostreptococcaceae* (24 % of *Clostridiales* clones), whereas in HC diet samples, the vast majority of clones (99 % of *Clostridiales* clones) were affiliated with *Peptostreptococcaceae*. A distribution similar to that of the HC diet sample was observed in the HP diet sample, where 94 % of the *Clostridiales* clones were also classified into the family *Peptostreptococcaceae* (Figure 2).

In the HP diet samples, *Fusobacteriales* was the most prevalent order (62 % of clones, Figure 2). Interestingly, only one representative of *Fusobacteriales* was found in HC diet samples, whilst no members of this order were discovered in DC diet samples. The sequences belonging to the order *Fusobacteriales* showed the closest similarity with *Fusobacterium varium* and *Fusobacterium mortiferum*.

In addition to the members of *Clostridiales*, 14 % of the sequenced clones in the HC diet sample were affiliated with the order *Erysipelotrichales* (Figure 2), which is classified into the same phylum as *Clostridiales*. More specifically, these sequences belong to Clostridial Cluster XVIII. By contrast, only one sequence that classified into the order *Erysipelotrichales* was discovered in HP diet samples (Figure 2).

In the DC diet, the sequence diversity was generally higher than in the HC and HP diets, as the sequences were classified into five different orders, two of which, *Lactobacillales* and *Bacteroidales,* were completely absent from the samples from the HC and HP diets (Figure 2). At the family level, all *Lactobacillales* sequences associated the *Streptococcaceae* and *Bacteroidales* sequences with *Prevotellaceae*.

### **Phylogenetic analysis of fraction 10 sequences**

**Figure 3:**
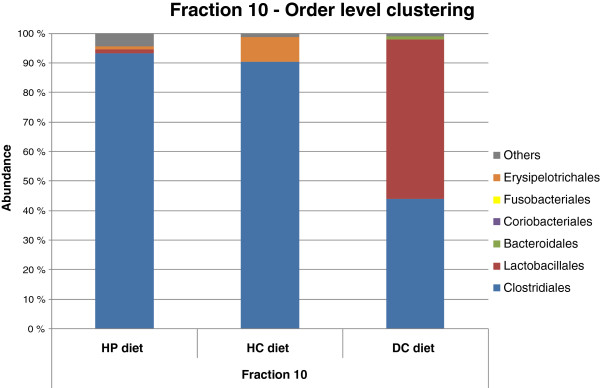
Phylogenetic analysis of fraction 10 sequences with DC, HP and HC diets.

Ninety-six sequences per sample were obtained from %G + C fraction 10. Sequences that affiliated with *Clostridiales* dominated in the DNA obtained from canine faecal samples during HP and HC diet phases (93 % and 90 % of all clones, respectively). At family-level classification, the *Clostridiales* sequences in the HP diet sample were allocated mainly to *Lachnospiraceae* (57 % of *Clostridiales* clones), *Peptostreptococcaceae* (35 % of *Clostridiales* clones) and *Ruminococcaceae* (6 % of *Clostridiales* clones). On the other hand, the majority of *Clostridiales* sequences in the HC diet sample (96 % of *Clostridiales* clones) were classified into the family *Peptostreptococcaceae* (Figure 3). A total of 85.5 % of these sequences were affiliated with *Clostridium hiranonis*, a member of Clostridial Cluster XI.

The abundance of *Clostridiales* sequences (44 %; mainly members of the family *Lachnospiraceae*) in the DC diet sample was smaller than in the HP and HC diet samples (Figure 3, p < 0.01). The most dominant group of bacteria in canine faecal samples with the DC diet in fraction 10 was *Lactobacillales*, more specifically *Streptococcaceae*, which comprised 54 % of the clones sequenced. Only two *Streptococcaceae* clones were found in HP diet samples, and no lactic acid bacteria were detected in HC diet samples (Figure 3).

### **Phylogenetic analysis of fraction 14 sequences**

**Figure 4:**
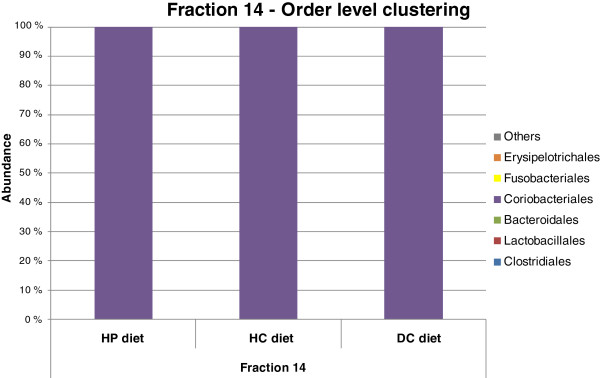
Phylogenetic analysis of fraction 14 sequences with DC, HP and HC diets.

Forty-eight sequences per sample were obtained from %G + C fraction 14. All 141 clones from the high %G + C fraction 14 that yielded a sequence of adequate quality were affiliated with the order *Coriobacteriales* (Figure 4). At the genus level, the majority of clones (n = 138) appeared to belong to *Collinsella* spp., with the remaining clones representing *Slackia* spp. and *Eggerthella* spp. Surprisingly, members of the order *Bifidobacteriales* were not discovered in any of the three samples (Figure 4).

**Figure 5:**
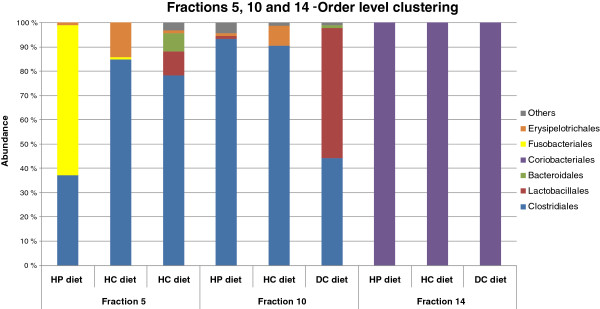
Phylogenetic analysis of fraction 5, 10 and 14 sequences with DC, HP and HC diets.

## Discussion

The influence of dietary animal-derived proteins and carbohydrates on canine intestinal microbiota was investigated. The %G + C profiles, as well as order-level sequence distribution in fraction 5, between the DC and HC diet samples did not differ considerably, most likely indicating that the modulatory influence of the HC diet on canine fecal microbiota is smaller than that of the HP diet when compared with the DC diet phase of the trial. This result is not surprising since both DC and HC diets consisted mainly of carbohydrate-rich components.

The amount and type of fermentable carbohydrates reaching the colon are primary factors influencing the abundance and variety of the resident bacterial population. The bacteria that can most rapidly degrade and use the digesta will proliferate beyond the others [23]. Corn starch, which was also included in our diets, has a high small intestinal digestibility and is therefore not expected to reach the large intestine in high amounts [24]. However, the DGGE band patterns obtained in a previous study indicated that this carbohydrate affected the composition of faecal bacteria in rats [9]. Therefore, the passage of corn starch into the large intestine might have been one of the reasons for sequence differences between DC and HP diet samples as well as between HC and HP diet samples.

The sequence diversity in the DC diet sample was generally higher than in the HC and HP diet samples, as the sequences were classified into five different orders, two of which, *Lactobacillales* and *Bacteroidales,* were completely absent in faecal samples from the HC and HP diets. The higher diversity was most likely due to the different ingredients of the DC diet sample, and the combination of corn and processing conditions might have resulted in a more versatile spectrum of fermentable substrates for various bacterial types [24].

In fraction 5 of the HP diet sample, the most abundant sequences belonged to the order *Fusobacteriales* and showed close similarity with the species *F. varium* and *F. mortiferum*. *F. variumF. necrophorumF. nucleatum* and *F. equinum* have been found to play roles in the pathogenesis of colonic, oropharyngeal, gingival, periodontal and other inflammatory processes, such as abscesses, pneumonia and sinusitis [25-27]. Given that the HP diet led to diarrhoea for all dogs in our study (data not shown), it could be hypothesized that species from the order *Fusobacteriales* could have caused the loss in faecal consistency, together with the high collagen concentration in the HP diet. The Greaves-meal diet, having a high digestibility, is known to soften the faeces, increase *Clostridium perfringens* levels and decrease bifidobacteria in dogs [28,29]. To our knowledge, *F. varium* and *F. mortiferum* have not been previously detected in canine faecal samples. Further characterization of isolates of these species should clarify whether they are commensals or opportunistic pathogens, or both, which is the case in the human intestine [27].

The results obtained from fraction 10 again indicate that the increased sequence diversity with the DC diet relative to the HP and HC diets was most likely due to the more versatile nutrient composition. In the HC diet sample, the most abundant sequences belonged to the order *Clostridiales*, showing the closest similarity with *Clostridium hiranonis*, which has been discovered previously in the canine GI tract and is considered to belong to the normal canine intestinal microbiota [12].

In fraction 14, all sequences in faecal samples of all dietary groups belonged to the order *Coriobacteriales*, suggesting that members of *Coriobacteriaceae* may be indicators of a healthy GI microbiota. For instance, in humans a high abundance of *Collinsella aerofaciens* has been associated with a lowered risk of colon cancer and inflammatory bowel disease [30,31]. To our knowledge, the presence of bacteria belonging to the order *Coriobacteriales* in canine faecal samples has been reported only in a recent 16 S rRNA gene sequencing study [6]. The order *Coriobacteriales* within the phylum *Actinobacteria* was found to be more abundant than previously estimated with conventional sequencing studies also in human faecal samples [22]. This is most likely due to the sequencing studies having been carried out without %G + C fractioning. It is evident that fractionating the total faecal DNA preparations minimizes PCR and cloning-derived bias, which is common in multi-template sequencing studies. In other words, fractionating facilitates the amplification and subsequent cloning of species with high G + C contents from diverse microbial communities [19,20].

No bifidobacteria were found, consistent with an earlier study [11]. However, contradictory data also exist, as many studies have found bifidobacteria in dogs [12,32]. Possibly, bifidobacteria were not part of the predominant intestinal microbiota of the Beagle dogs participating in our study. Another potential explanation for this unanticipated result may be that the universal 16 S rRNA gene-targeted primer pair contained mismatches to many bifidobacterial species, which could have led to significant underestimation of bacteria belonging to this genus.

*Clostridiales* and *Coriobacteriales* were the most prevalent bacterial orders in the faecal samples of all dietary groups. Suchodolski and coworkers [13], by contrast, reported that *Fusobacteriales* and *Bacteroidales* were the most representative orders in the canine colon. It is noteworthy, however, that we analysed only three %G + C fractions, which showed the most pronounced alterations between the dietary groups. Our aim was not to obtain an overall picture of the canine faecal microbiota, but to elucidate the diet-derived effects on the microbial community structure in the lower intestine.

## Conclusions

Significant dietary effects on canine intestinal microbiota were detected. The DC diet sample showed a high abundance of representatives of the orders *Clostridiales*, *Lactobacillales* and *Coriobacteriales* and the presence of representatives of the order *Bacteroidales*. Sequence diversity was higher with the DC diet sample, as representatives of the orders *Lactobacillales* and *Bacteroidales* were not detected in the HP and HC diet samples. During feeding of the HC and HP diets the representatives of Clostridial Cluster XIVa were suppressed in canine faecal samples. The HC diet favoured representatives of Clostridial Cluster XVIII. The HP diet favoured representatives of the order *Fusobacteriales*, which could play a role in induction of diarrhoea together with the lower carbohydrate concentration entering the large intestine.

## Methods

### **Animals and diets**

Five beagle dogs (origin: Harlan-Winkelmann, Borchen, Germany; age: 5 years; body weight: 18–22 kg; sex: male) from the Experimental Animal Unit of Helsinki University, Finland, were assigned to this study. The experimental protocol was approved by the local Ethics Committee for Animal Use and Care in Helsinki, Finland (license no. ESLH-2008-04002/Ym-23). The dogs were housed individually indoors and were vaccinated and dewormed 6 months and 2 months before the trial, respectively.

The study was designed as an incomplete Graeco-Latin square in which the following six trial phases were included: baseline phase (DC diet: Mastery Pro Adult Dog Maintenance, Raili Pispa Oy, Muurla, Finland; crude protein: 264 g/kg, starch: 277 g/kg, 14 d), diet phases (HP diet with a high collagen content: crude protein: 609 g/kg, starch: 54 g/kg; HC diet: crude protein: 194 g/kg, starch: 438 g/kg; and DC diet, 21 d each), and washout phase W HP and W HC (DC diet after the HP and HC diets, respectively, 28 d each) (Tables 1 and 2).

**Table 1:** Composition of the HP, HC and DC diets fed to five dogs in a Graeco-Latin square design

**Ingredient**	**HP diet**	**HC diet**	**DC diet**
Greaves meal	80 %	17 %	0 %
Dehydrated meat	0 %	0 %	27 %
Corn flakes (heat-treated)	15 %	72 %	0 %
Maize (cooked)	0 %	0 %	65 %
Sunflower oil	2 %	8 %	0 %
Vegetable oils	0 %	0 %	3 %
Minerals and vitamins	3 %	3 %	5 %
Total	100 %	100 %	100 %

**Table 2:** Nutrient analysis and trace elements of the HP, HC and DC diets fed to dogs in a Graeco-Latin square design

**Nutrient**	**g/kg**		
	**HP diet**	**HC diet**	**DC diet**
Dry matter	930.4	906.9	913.9
Crude ash	49.0	39.4	85.4
Crude protein	609.1	193.7	263.5
Crude fat	150.4	132.7	99.7
Crude fibre	73.8	59.0	103.8
Starch	54.4	438.4	277.0
**Mineral**	**g/kg**		
Calcium	6.6	6.2	16.4
Sodium	7.0	4.8	6.0
Magnesium	1.5	1.3	1.3
Potassium	5.2	2.2	5.8
Phosphorus	6.2	4.4	12.7
**Trace element**	**mg/kg**		
Copper	23.4	19.2	21.6
Iron	252.1	196.3	365.2
Zinc	135.2	108.1	205.1
Manganese	101.0	277.1	111.3

The HP and HC diets were formulated at the Institute of Animal Nutrition (Freie Universität Berlin, Berlin, Germany) to obtain considerable differences between them, and were analysed according to previously developed standard methods for feed analyses [33]. The animals were fed twice daily, at 8 a.m. and 3 p.m. The metabolic energy content was 1.54 MJ/100 g of the HP diet, 1.49 MJ/100 g of the HC diet and 1.25 MJ/100 g of the DC diet. The diets were given to meet the daily energy requirements estimated at 0.5 MJ metabolisable energy/kg^0.75^. Water was provided freely during the entire study.

### **Sample collection and handling**

All dogs defecated within one hour of the morning feed, and fresh naturally-passed faeces were collected for sampling immediately after defecation. Faecal samples were taken on three consecutive days at the end of each dietary phase: for the baseline phase on days 10–12, for diet phases on days 15–17 and for washout phases on days 22–24. All dogs were housed individually and faeces were collected immediately after defecation to avoid coprophagia. Faecal samples were collected from the floor, leaving the bottom layer untouched to ensure that the sample contained only faecal material. The rest of the faeces was collected for disposal. Each animal received all three diets (HP, HC and DC). The total number of samples was 90 from five dogs, as we took the faecal sample on three consecutive days at the end of each of the six diet phases (baseline, HP, HC, DC, 2 washouts). Since we were mostly interested in changes during the three diets given, we used only the samples taken on three consecutive days at the end of the DC, HP and HC diet phases; therefore, the number of samples was 45 from five dogs (3 samples/3 days for 5 dogs). The samples were thoroughly homogenized and 1-g aliquots were immediately placed in pre-weighed sterile Sarstedt faecal collection tubes with a spatula (Sarstedt Oy, Vantaa, Finland) and frozen at −80 °C until further analysis. To increase the amount of faecal DNA needed for the preparative separation, the three samples taken on three consecutive days from each dog (n = 5) were thawed and pooled prior to DNA extraction. Therefore, the number of samples subjected to DNA extractions was 15.

### **Bacterial DNA extraction from faecal samples**

Bacterial DNA extraction was carried out essentially as described earlier [34]. Briefly, bacteria in the samples were initially washed and separated by repeated differential centrifugation to remove solid particles and inhibitory factors (e.g., complex polysaccharides), which disturb the subsequent DNA purification process and the downstream molecular applications. Bacterial cell walls were then disrupted using both enzymatic and mechanical lysis steps, and finally the chromosomal DNA was quantitatively purified by gravity-flow anion exchange tips.

### **Total community analysis by %G + C profiling**

The faecal microbial DNA of each dietary group was pooled (samples from all five dogs after DC, HC and HP diets). The pooled DNA samples were concentrated with isopropanol precipitation and dissolved in 400 μl of TE buffer, after which the DNA concentration was determined with a UV spectrophotometer prior to the %G + C profiling.

In %G + C profiling, each of the three pooled DNA samples was fractionated by 72-h CsCl equilibrium density gradient ultracentrifugation (100 000 × g), which separates chromosomes with different G + C content. This separation is based on differential density imposed by the AT-dependent DNA-binding dye bis-benzimidazole [35]. In the following ultracentrifugation, the formed gradients were pumped through a flow-through UV absorbance detector set to 280 nm and %G + C fractions were collected at 5 % intervals.

Three DNA fractions (referred to herein as fractions 5, 10 and 14) from each sample were subjected to desalting with PD-10 columns (GE Healthcare Life Sciences, Little Chalfont, Buckinghamshire, UK) for subsequent 16 S rDNA gene PCR amplification with a universal broad-range primer pair.

### **Amplification of the 16 S rRNA genes and sequencing**

The nearly complete 16 S rRNA gene fragments from each of the three desalted DNA fractions were amplified with end-point PCR using a universal primer pair corresponding to *Escherichia coli* 16 S rRNA gene positions 8–27 and 1389–1405, with sequences 5'-AGAGTYYGATCCTGGCTCAG-3' [36] and 5'-TGACGGGCGGTGTGTAC-3' [37], respectively. The oligonucleotide primers were synthesized commercially by MWG-Biotech AG, Ebersberg, Germany. The 50-μl PCR reactions contained 1 × DyNAzyme™ Buffer (Finnzymes, Espoo, Finland), 0.2 mM of each dNTP, 20 pmol of primers, 1 U of DyNAzyme™ II DNA Polymerase (Finnzymes, Espoo, Finland), 0.125 U of Pfu DNA polymerase (Fermentas*,* Vilnius*,* Lithuania) and 10 μl of desalted fractioned DNA template (1:10 dilution from the desalted stock solution). PCR amplification was carried out with 30 cycles for each fraction. After the PCR reaction, the correct size of amplification products was verified on an ethidium bromide stained agarose gel. Finally, the PCR products were purified with the QIAquick PCR Purification Kit (Qiagen, Hilden, Germany) and cloned and sequenced with Agowa genomics (Berlin, Germany).

### **Data handling and statistical analyses**

The %G + C content represented by each gradient fraction was determined by linear regression analysis (*r*^2^ > 0.99) of data obtained from the control gradients containing bacterial standard DNA samples of known %G + C composition (*Clostridium perfringens*, *Escherichia coli* and *Micrococcus lysodeikticus*). Two sample t-tests were used to detect significant differences between the %G + C profiles of different treatment groups.

In the analysis of 16 S rDNA sequences, the bidirectional contigs were checked for orientation and sequence quality, and only the ones with correct primer sequences and one-way read length above 900 bp were accepted for further analyses. Potential chimeras were revealed using Ribosomal Database Project II Chimera Check. The PCR primer and cloning vector sequences were removed, and 16 S rDNA fragments were compared with a public 16 S Ribosomal Database Project II (RDP-II) to determine the closest match to aligned sequences of known species. If the S_ab_ score (similarity score *a* versus *b*) of a cloned sequence was over 0.95 of the strain type of any known species, the cloned sequence was assigned to that species [38].

The microbial community comparison based on 16 S rRNA sequences was performed using Library Compare tool of RDP II. This tool uses the RDP naïve Bayesian classifier to provide rapid classification of library sequences into the new phylogenetically consistent higher-order bacterial taxonomy. It estimates the probability of observing the difference in a given taxon for the two libraries using a statistical test [34].

## Abbreviations

16 S rRNA: 16 S Ribosomal RNA; PCR: Polymerase Chain Reaction; G-C: Guanine-Cytosine; 16 S rDNA: 16 S Ribosomal DNA; DGGE: Denaturing Gel Gradient Electrophoresis; TE: Tris-EDTA (Ethylenediaminetetraacetic acid); UV: UltraViolet; CsCl: Caesium Chloride; AT: Adenine-Thymine; dNTP: DeoxyriboNucleotide Triphosphate; Pfu: Pyrococcus Furiosus; bp: Base Pair.

## Authors` contributions

IH, WMV, JH, JZ and TS conceived the study design; IH performed sample collection; TR and JA performed bacterial DNA extraction, %G + C profiling and sequencing analysis; AK and SA performed statistical analysis; all authors contributed to the writing of the manuscript.
